# Chyle leakage in port incision after video-assisted thoracoscopic surgery: case report

**DOI:** 10.1186/1749-8090-5-83

**Published:** 2010-10-15

**Authors:** Lin Ma, Qiang Pu, Yunke Zhu, Lunxu Liu

**Affiliations:** 1 Department of Thoracic Surgery, West China Hospital, Sichuan University, Chengdu 610041, China

## Abstract

A 26-year-old Asian male was found to have chyle leakage from the port incision after video-assisted thoracoscopic surgery (VATS) for excision of pulmonary bullae. The diagnosis was confirmed by oral intake of Sudan black and by lymphoscintigraphy. The leakage resolved after 5 days of restricted oral intake and total parenteral nutrition. No leakage recurred after return of oral intake. Possible explanations for the port incision chyle leakage are obstruction of the thoracic duct, which induced retrograde drainage of the lymphoid fluid, or an aberrant collateral branch of the thoracic duct in the chest wall.

## Background

Chylous effusion is not a rare complication of thoracic surgery. Cerfolio et al. [1] reported that 47 of 11351 patients who received thoracic operations experienced chylothorax complications. In these 47 cases, 27 had undergone esophageal operations, 13 lung operations, 6 mediastinal operations, and 1 underwent surgery of the thoracic aorta due to an aneurysm. In China, Zhao et al. [2] reported that of 4084 patients who had undergone resections due to lung cancer, 12 developed chylothorax complications. In addition, the authors reported that of 4479 cases of resection due to esophageal cancer, 52 patients developed chylothorax complications. Thus, the incidence of postoperative chylothorax in patients who underwent surgery for lung cancer was 0.29%, and that of esophageal cancer was 1.16%. Chylothorax causes serious clinical consequences including cachexia and immunodeficiency [3]. Chyle leakage in port incisions has rarely been reported. Chyle leakage can be confirmed by qualitative testing for the presence of chyle, the Sudan black test, and by dynamic lymphoscintigraphy.

## Case presentation

**Figure 1:**
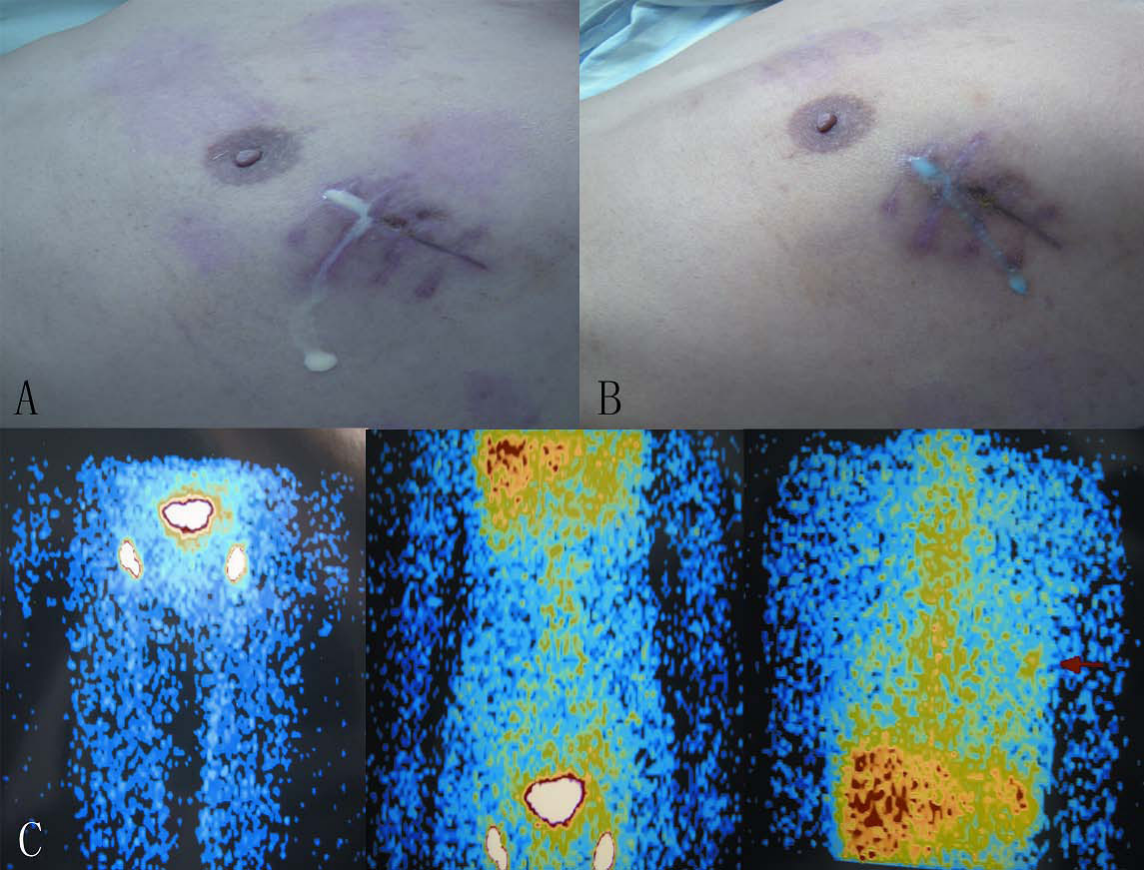
**Chyle leakage in port incision in the left chest wall and its lymphoscintigraphy**. A: Milky white and odorless liquid leaked from the front of the major port incision. B: The leakage became blue after the patient ingested Sudan Black. C: Lymphoscintigraphy using Tc-99 m sodium phytate as a tracer. The image was taken 60 min after injection. An abnormal tracer accumulation was evident in the left chest wall corresponding to the major incision (red arrow). No tracer accumulation was found in the pleural cavity.

A 26-year-old Asian male underwent video-assisted thoracoscopic surgery (VATS) for excision of bullae because of recurrent left spontaneous pneumothorax. The thoracoscope access port was located at the midaxillary line of the 7^th ^intercostal space and was 1.5 cm in length. The major port incision was on the anterior axillary line of the 3^rd ^intercostal space and was 4 cm in length. No adhesions were present in the pleural cavity. Two bullae were found at the apex of left lung and were resected with an endostapler without complications. Three days after surgery, milky, odorless liquid was noted leaking from the front of the major port incision (Figure 1A) at a rate of 50 ml/d. A qualitative test for chyle was positive. Microscopic examination revealed monocytes (750 × 10^6 ^cells/L) and erythrocytes (450 × 10^6 ^cells/L), but no neutrophils. After the patient ingested Sudan black, the leakage turned blue (Figure 1B). A diagnosis of chyle leakage from the incision was thus made. Dynamic lymphoscintigraphy was performed after intradermal injection of Tc-99 m sodium phytate in each foot. Approximately 60 min after injection, tracer accumulation in the bilateral inguinal lymph nodes was captured. Abnormal tracer accumulation was detected in the major port incision of the left chest wall; however, no tracer accumulation was detected in the pleural cavity, and no other nearby collateral lymphatic branch was revealed within the chest wall (Figure 1C).

Because the leakage persisted, 2 weeks after surgery debridement of the incision was performed. Biopsy of the tissue at the incision was performed, and the incision was carefully sutured. The biopsy showed striated muscle. Despite the surgical treatment, the leakage continued. Oral intake was restricted and total parenteral nutrition was administered (20 d after the first operation), and the leakage ceased after 5 days. The therapy was continued for another 3 days, after which oral intake was resumed. The leakage did not reappear.

## Discussion

Although there have been many reports of postoperative chylothorax after thoracic surgery, there have been no reports on chyle leakage from chest wall incisions. To our knowledge, this is the first report of chyle leakage from a chest wall incision. The diagnosis of chyle leakage was confirmed by qualitative testing for chyle and the Sudan black test.

The normal flow rate in the thoracic duct is 1500-2000 ml/d. In our case, the quantity of the leakage was 50 ml/d while the patient was receiving a normal diet. We assume that an abnormal duct in the chest wall which drained chyle was injured in the VATS port placement. Injury to this abnormal duct might have resulted in retrograde drainage of chyle due to an obstruction in the thoracic duct, or because of an aberrant collateral branch of the thoracic duct in the chest wall.

It has been reported that when the thoracic duct or vena cava is obstructed, abnormal tracer accumulation can be detected by lymphangiography in the intercostal, pulmonary, and pleural lymphatic vessels [4]. Moreover, another study reported that the pulmonary lymph nodes can be detected even if the thoracic duct does not undergo any pathological changes [5]. At present, lymphoscintigraphy is considered the best noninvasive method of examination of the lymphatic system. When Tc-99 m sulfur colloid is used as the tracer, the lymphatic vessels and lymph nodes are clearly exhibited [6]. Because Tc-99 m sulfur colloid is not available in our hospital, we used Tc-99 m sodium phytate. Only the inguinal lymph nodes and abnormal accumulation of the tracer in the left chest were revealed. The thoracic duct and other lymphatic vessels were not exhibited with this tracer; thus, whether there was blockage of thoracic duct or the existence of an aberrant collateral branch of the thoracic duct remained undetermined.

## Conclusions

This report presented a rare and previously unreported occurrence of chyle leakage. Lymphoscintigraphy would be the appropriate choice for diagnosis and precise localization of leakage in patients with postoperative chylothorax, spontaneous chylothorax, or other chyle leakage.

## Competing interests

The authors declare that they have no competing interests.

## Authors' contributions

LM was involved in drafting the manuscript. QP was involved in acquisition of data. YZ was involved in preparing the figures. LL designed and revised the manuscript. All authors have read and approved the final manuscript.

## Consent

Written informed consent was obtained from the patient for publication of this case report and any accompanying images. A copy of the written consent is available for review by the Editor-in-Chief of this journal.

## References

[B1] Cerfolio RJ Allen MS Deschamps C Trastek VF Pairolero PC Postoperative chylothorax J Thorac Cardivasc Surg 1996 112 1361 1365 10.1016/S0022-5223(96)70152-6 8911335

[B2] Zhao J Zhang DC Wang LJ Clinical features of postoperative chylothorax for lung cancer and esophageal cancer Chin J Surg 2003 41 47 49 12760759

[B3] Talwar A Lee HJ A contemporary review of chylothorax Indian J Chest Dis Allied Sci 2008 50 343 351 19035053

[B4] Jose Restrepo M Vicente Caride J Lymphoscintigraphy and Radionuclide Venography in Chylothorax Clin Nucl Med 2004 29 440 441 10.1097/01.rlu.0000129125.05486.c5 15192469

[B5] Clark RA Colley DP Pulmonary lymphatics visualized during pedal lymphangiography Radiology 1980 136 29 32 7384519 10.1148/radiology.136.1.7384519

[B6] Pui MH Yueh TC Lymphoscintigraphy in chyluria, chyloperitoneum and chylothorax J Nuc Med 1998 39 1292 12966 9669413

